# Transcriptome analysis of genes involved in defense against alkaline stress in roots of wild jujube (*Ziziphus acidojujuba*)

**DOI:** 10.1371/journal.pone.0185732

**Published:** 2017-10-04

**Authors:** Mingxin Guo, Shipeng Li, Shan Tian, Bei Wang, Xusheng Zhao

**Affiliations:** College of Life Science, Luoyang Normal University, Luoyang, Henan, China; Institute of Genetics and Developmental Biology Chinese Academy of Sciences, CHINA

## Abstract

Wild jujube (*Ziziphus acidojujuba* Mill.) is highly tolerant to alkaline, saline and drought stress; however, no studies have performed transcriptome profiling to study the response of wild jujube to these and other abiotic stresses. In this study, we examined the tolerance of wild jujube to NaHCO_3_-NaOH solution and analyzed gene expression profiles in response to alkaline stress. Physiological experiments revealed that H_2_O_2_ content in leaves increased significantly and root activity decreased quickly during alkaline of pH 9.5 treatment. For transcriptome analysis, wild jujube plants grown hydroponically were treated with NaHCO_3_-NaOH solution for 0, 1, and 12 h and six transcriptomes from roots were built. In total, 32,758 genes were generated, and 3,604 differentially expressed genes (DEGs) were identified. After 1 h, 853 genes showed significantly different expression between control and treated plants; after 12 h, expression of 2,856 genes was significantly different. The expression pattern of nine genes was validated by quantitative real-time PCR. After gene annotation and gene ontology enrichment analysis, the genes encoding transcriptional factors, serine/threonine-protein kinases, heat shock proteins, cysteine-like kinases, calmodulin-like proteins, and reactive oxygen species (ROS) scavengers were found to be closely involved in alkaline stress response. These results will provide useful insights for elucidating the mechanisms underlying alkaline tolerance in wild jujube.

## Introduction

Salinity and alkalinity are the two main environmental factors that inhibit the growth and development of crops, thus reducing agricultural production, and, in severe conditions, resulting in plant death. An estimated 950 million hectares of the global land surface consists of saline-alkaline soil [1]. Previous studies have demonstrated that saline and alkaline stresses have different impacts on plant [2]. Saline soils, which are generally dominated by the cation Na^+^, and the anions Cl^−^ and SO4^2-^ result in osmotic stress and ion injury. Alkaline stress (NaHCO_3_ and Na_2_CO_3_), involves multiple factors, including osmotic stress and ion injury, and the elevated soil pH (pH > 8.5) is much more destructive to plants than neutral salts [3].

Alkaline stress can also inhibit plant growth by decreasing nutrient solubility. At the high pH of alkaline soil, metal ions, especially iron (Fe), are sparingly soluble and are not available to plants [4]. Researchers have unveiled key molecular components including iron transporters, iron reductases, and enzymes involved in phytosiderophore biosynthesis. Manipulation of these molecular components has produced transgenic plants with enhanced tolerance to alkaline stress on calcareous soils [4–6]. For example, ectopic expression of the barley nicotianamine aminotransferase, HvNAAT, or the yeast Fe^3+^-chelate-reductase gene refre1/372 exhibited an enhanced tolerance to low-Fe availability and higher grain yield in calcareous soil [4,5].

Many other molecular mechanisms and regulatory networks are involved in plant tolerance to alkaline stress. For example, comprehensive transcriptional profiling of alkaline-tolerant plants, such as *Puccinellia tenuiflora* [7], *Leymus chinensis* [8], and *Tamarix hispida* [9] grown under NaHCO_3_ or Na_2_CO_3_ treatments revealed that batches of genes of different functional categories were differentially expressed, indicating there are complex molecular mechanisms and regulatory networks in plants. In addition, some alkaline tolerant or sensitive mutants, such as *pks5*, *j3*, *alt1* have been isolated and characterized [10–12]. In Arabidopsis, chaperone J3 positively regulates alkaline tolerance by interacting with protein kinase PKS5 to increase plasma membrane H^+^-ATPase activity [10,11]. In rice, ALT1, a putative Snf2 family chromatin remodeling ATPase, functions in alkaline tolerance through enhanced defense against oxidative stress in rice [12].

Wild jujube (*Ziziphus acidojujuba* Mill.) has a close morphological resemblance to jujube (*Ziziphus jujuba* Mill.) and is regarded as the direct ancestor of the cultivated species. Wild jujube was usually used as rootstock for grafting and provide important traits for improvement of jujube. Most of wild jujube is shrub with smaller fruit, leaf than cultivated jujube. The kernel of wild jujube was round or oval shape, containing one or two seeds which were used as a traditional herbal medicine. More importantly, wild jujube exhibits strong tolerance to drought, salinity, and alkalinity and wider distribution in barren mountains, Gobi desert and other arid and semi-arid areas [13,14].

NaHCO_3_-NaOH buffer is a stable alkaline solution that can be adjusted from pH 9.0 to pH 11.5, making it an ideal buffer for testing alkaline stress. Previous studies have shown that pH 9.5 is a suitable pH value for alkaline treatment [12]. Solutions with pH above 10.0 cause serious and quick injury, while solutions that are not sufficiently alkaline make it difficult to study alkaline stress. How the transcriptomes of wild jujube plant roots respond to alkaline stress may help reveal the mechanism of alkaline tolerance in wild jujube.

In this study, the transcriptomes from roots of wild jujube treated with NaHCO_3_-NaOH solution (pH 9.5) for 0, 1, and 12 h were constructed, the transcript profiles in response to alkaline stress were generated, and potential candidate genes involved in alkaline stress response were identified. This study provides a foundation for elucidating the molecular mechanisms underlying tolerance to alkaline stress in wild jujube.

## Results

### Phenotypic and physiological features of wild jujube seedlings in tolerating alkaline stress

**Fig 1:**
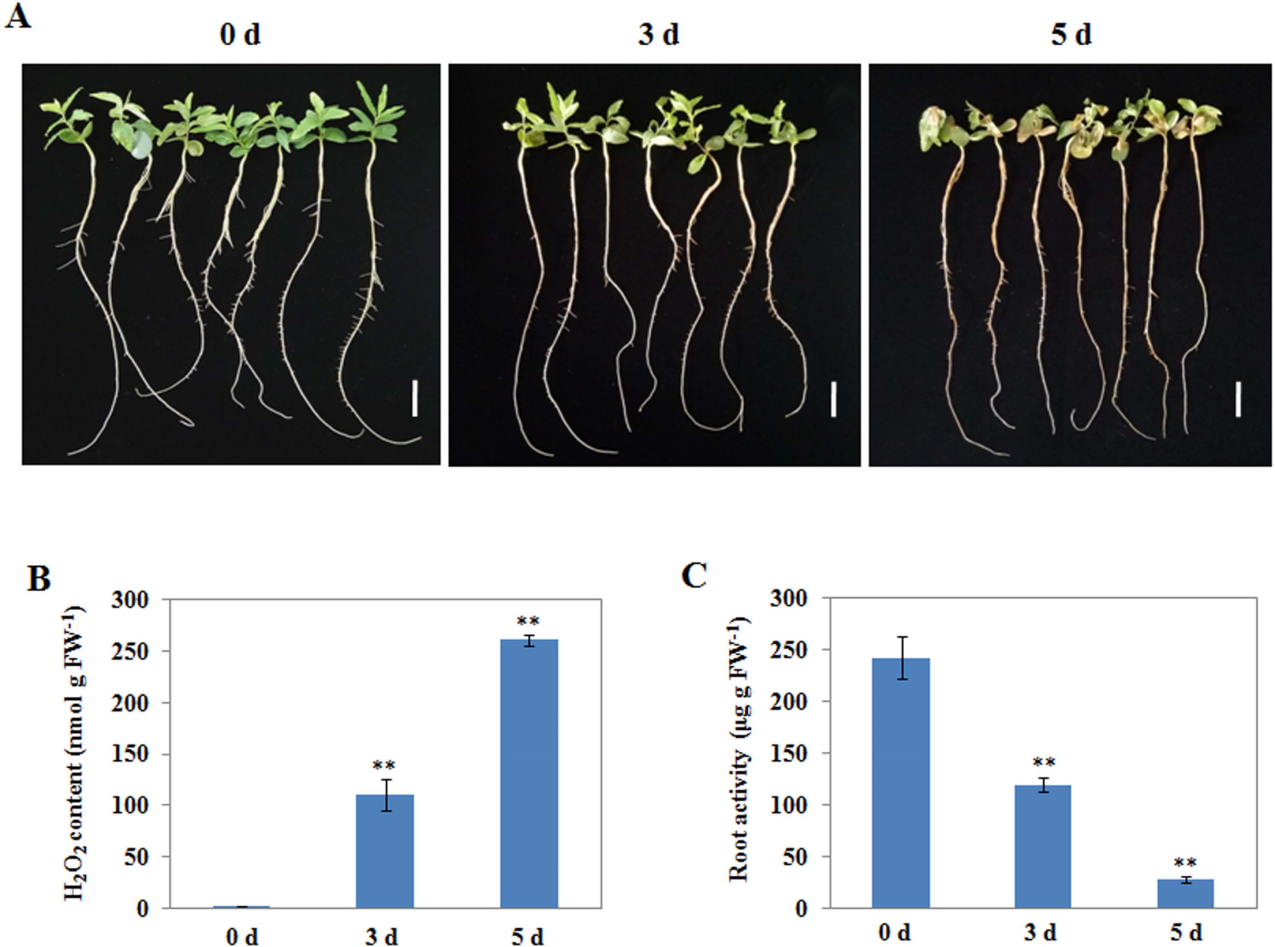
Physiologic characteristics of wild jujube under salt and alkaline-salt treatment. Seedlings at the six-leaf stage were subjected to an alkaline treatment (pH 9.5). (A) Time-course observation for tolerance phenotype at 3, and 5 d after the start of treatment. Bars = 2 cm. (B) Quantitative measurement of H_2_O_2_ in wild jujube leaves during pH 9.5 treatment. Values are means± SD (n = 3). (C) Quantitative measurement of root activity in wild jujube during pH 9.5 treatment. Values are means± SD (n = 3).

To obtain an overview of alkaline tolerance phenotype of wild jujube, six-leaf stage seedlings were subjected to treatment with NaHCO_3_-NaOH solution (pH 9.5). The seedlings grew normally at the beginning of the second day. However, the leaves of seedlings became withered 3 d after treatment, and wilted completely by 5 d (Fig 1A).

We examined whether the alkaline stress causes oxidative stress in wild jujube seedlings by quantifying the content of H_2_O_2_. It showed that the H_2_O_2_ content in leaves was increased significantly from 1.2 to 261.1 nmol g FW^-1^ during the alkaline stress of pH 9.5 treatment (Fig 1B). We further examined changes in root activity of seedlings under alkaline treatment of pH 9.5 solution. The root activity decreased quickly from 242.4 to 27.2 μg g FW^-1^ (Fig 1C).

### Illumina sequencing and reads assembly

To obtain a comprehensive understanding of alkaline-induced changes at the transcriptome level in wild jujube, six libraries of roots were created from seedlings exposed to pH 9.5 alkaline solution for 0, 1, and 12 h (two biological replicates for each time point). The raw reads generated from the each library ranged from 26,596,774 to 35,070,224. After removing the reads with low quality, the proportion of the clean reads were all above 95% for each library. Of all the clean reads from six libraries, 63.36–72.77% were mapped to genome, of which 87.91% were mapped to unique locations (Table 1). The reads from six libraries were combined and assembled into 32,758 non-redundant unigenes.

**Table 1:** Statistical analysis of wild jujube reads in six libraries mapped to the jujube reference genome.

Samples_ ID	Raw reads	Clean reads	Mapped reads	Mapped unique reads	Mapped multi reads	Mapping ratio (%)
0h_1	35,070,224	33,428,841	24,294,540	21,418,928	2,875,612	72.68
0h_2	28,561,312	27,436,896	19,966,716	17,620,832	2,345,884	72.77
1h_1	29,810,474	28,759,618	20,920,839	18,368,983	2,551,856	72.74
1h_2	34,111,020	33,047,480	22,060,855	19,450,795	2,610,060	66.76
12h_1	31,293,856	30,251,747	19,168,906	16,790,162	2,378,744	63.36
12h_2	26,596,774	25,418,305	16,220,107	14,192,897	2,027,210	63.81

### Identification of differentially expressed genes (DEGs)

**Fig 2:**
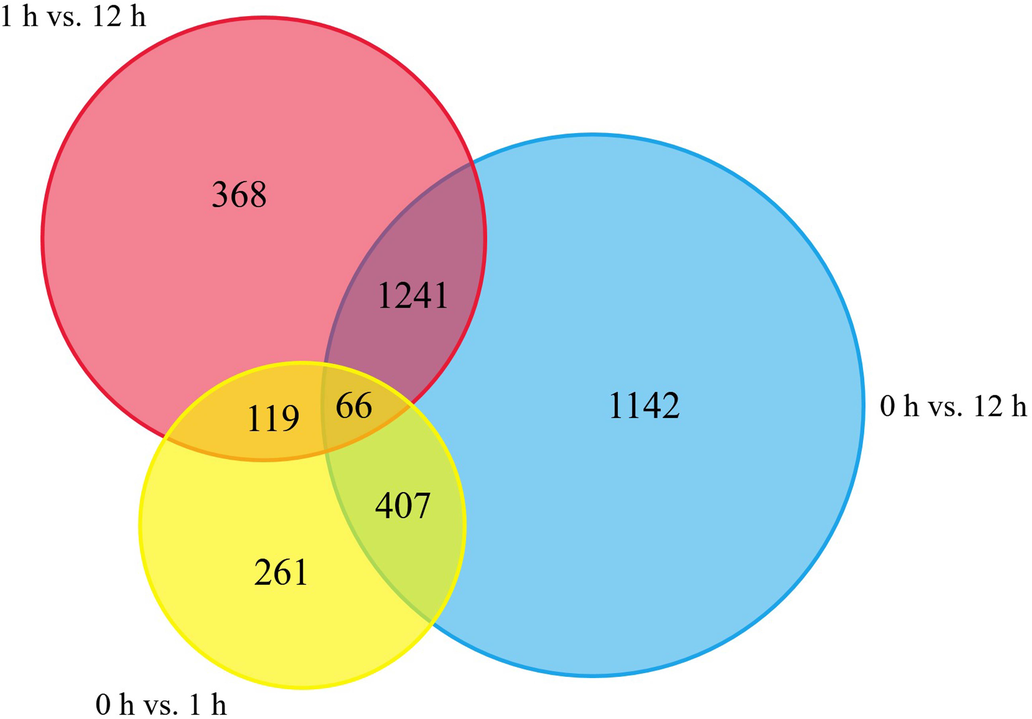
The overlap of differentially expressed genes (DEGs) in wild jujube (*Ziziphus acidojujuba*) roots under alkaline treatment. The three data sets compared 0 h vs. 1 h (yellow), 1 h vs. 12 h (red), and 0 h vs. 12 h (blue).

To investigate the patterns of gene expression under alkaline treatment, FPKM values were calculated for each sample to normalize the expression. Based on a threshold of *Q* value ≤ 0.05 and |log_2_(fold change)| ≥ 2, there were 853 and 2,856 genes differentially expressed after treatment for 1 and 12 h, respectively (S1 and S2 Tables). And a total of 1,794 DEGs were detected between 1 and 12 h (S3 Table), which presented 3,604 DEGs among 0, 1, and 12 h (S4 Table). The characteristics of the DEGs in wild jujube at different alkaline stress time points were shown in Table 2. The overlap of DEGs among the three time points was analyzed (Fig 2), and the results showed that only a tiny minority of number (66 genes) were found to be differentially expressed in the three data sets (0 vs. 1 h, 1 h vs. 12 h and 0 vs. 12 h).

**Table 2:** The characteristics of the DEGs in wild jujube at different alkaline stress time points.

Items	Number of genes		
	0 h vs. 1 h	0 h vs. 12 h	1 h vs. 12 h
Total	853	2856	1794
Up-regulated	735	1473	796
Down-regulated	118	1383	998

### Functional annotation of the DEGs

**Fig 3:**
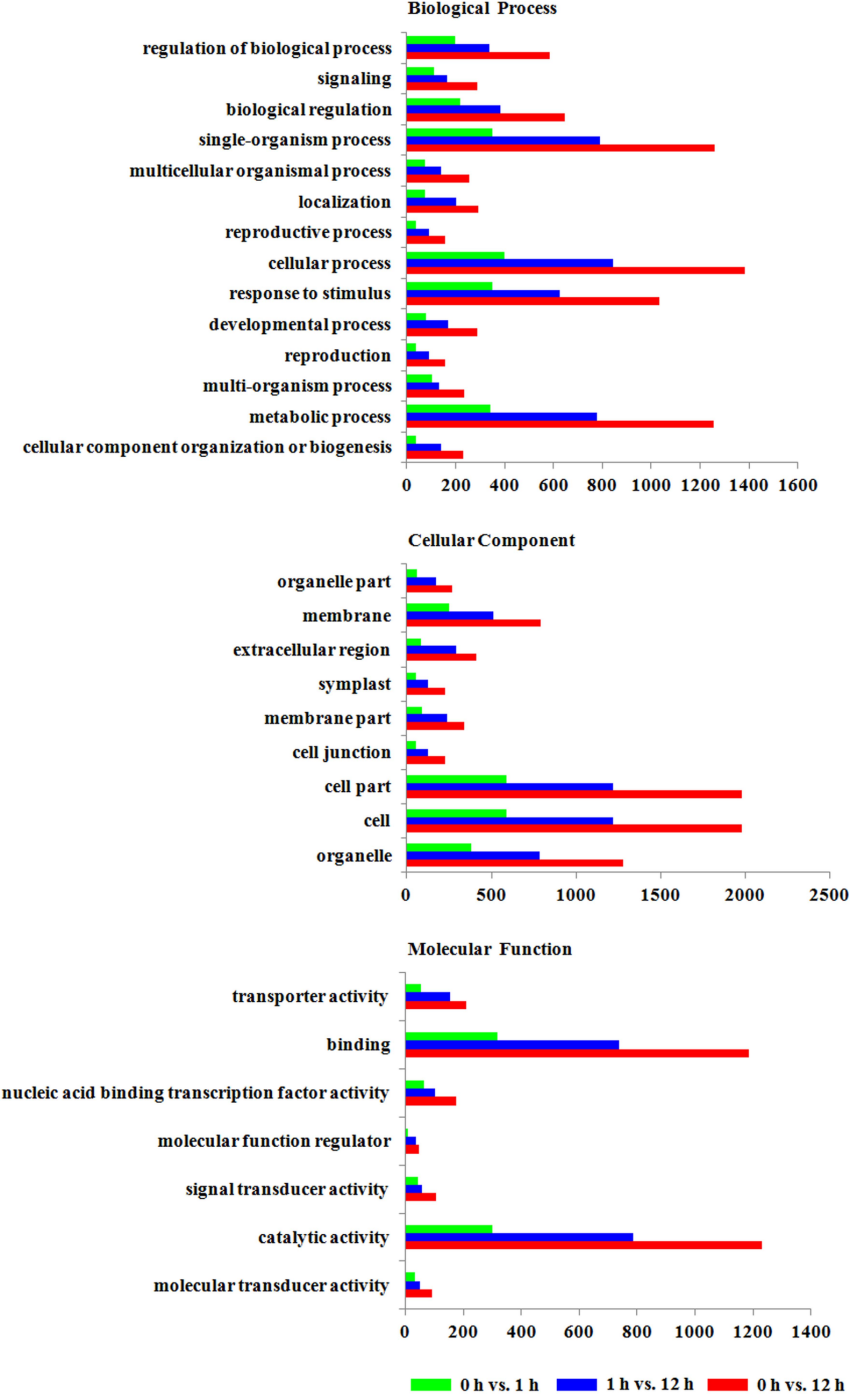
Gene ontology (GO) categories assigned to the DEGs in response to alkaline stress. The X- axis represents the gene numbers, and the Y- axis shows the GO subcategories.

To functionally annotate the DEGs in the three data sets (0 vs. 1 h, 1 h vs. 12 h and 0 vs. 12 h), we aligned all of the DEGs against the Gene Ontology (GO) and Kyoto Encyclopedia of Genes and Genomes (KEGG) database. For the GO analysis, the DEGs were annotated to three major GO categories: Biological Process (BP), Cell Component (CC) and Molecular Function (MF). The three data sets showed similar GO classification of the DEGs, the most abundant category was biological processes, followed by cell component and molecular functions (Fig 3 and S5 Table). In the biological process category, cellular process, single-organism process, response to stimulus, and metabolic process were the most abundant groups in the three data sets. In the cell component category, the three most abundant groups in the three data sets were entire cell, cell parts, and organelles. Similarly, in *Tamarix hispida* roots, entire cell, cell parts, and organelles were also most abundant groups under NaHCO_3_ stress [9]. For the molecular function category, the two most abundant groups in the three data sets were binding (average 38.48%) and catalytic activity (average 37.82%), which were much more abundant than other groups. Consistent with these results, binding and catalytic activity were also the two most abundant groups in maize and *T*. *hispida* after salt and NaHCO_3_ stress, respectively [9,15], which indicated binding and catalytic activity play important roles in stress response in plant roots.

KEGG analysis suggested that there were 129, 147, and 174 pathways in the three data sets involved in alkaline stress (S6 Table). Among these pathways, metabolic pathways and biosynthesis of secondary metabolites were the two largest categories in three data sets. The top 10 pathways possibly regulated by alkaline stress are presented in S7 Table.

### Validation of RNA-seq data by quantitative real-time PCR (qRT-PCR)

**Fig 4:**
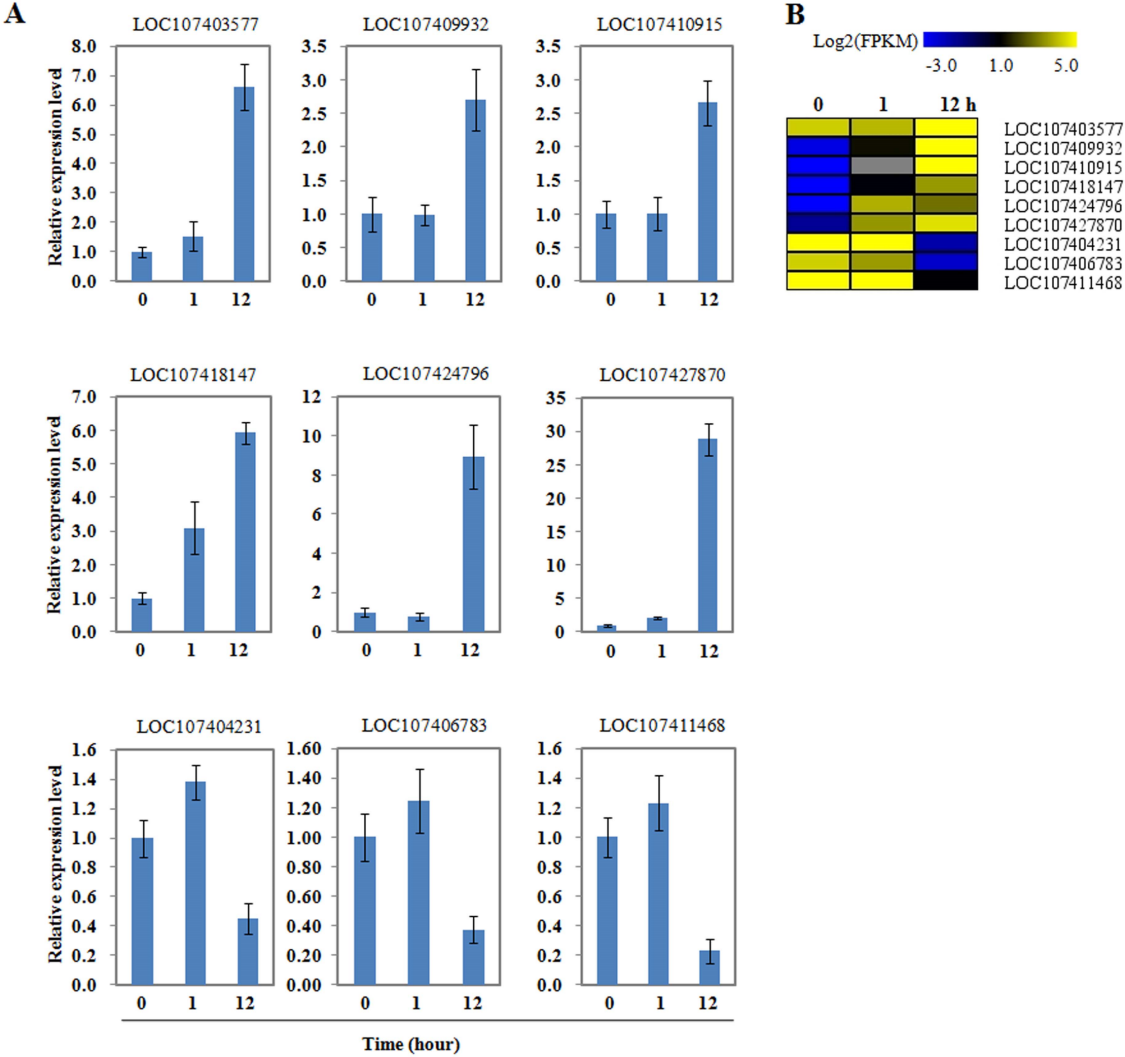
Expression analysis of selected genes. (A) Quantitative real-time PCR was conducted on the roots of hydroponically grown seedlings of wild jujube at the six-leaf stage. *ZaActin* was used as an internal control. Data shown are mean values of three biological replicates with SD. (B) The heat map was constructed based on log_2_ FPKM values at different time points. The color scale is shown at the top.

To verify the RNA-seq transcriptome data, nine genes were selected, six that were up-regulated genes and three that were down-regulated. The expression levels of the selected genes were quantified using qRT-PCR, and the results were highly in agreement with RNA-seq results (Fig 4).

### Screen alkaline stress- related candidates according to the transcriptome profile

#### Transcriptional factors (TFs)

In this study, many TFs, such as ERF, bHLH, DREB, WRKY, MYB, NAC, zinc finger, and bZIP were differentially expressed when wild jujube plants were exposed to alkaline stress (S8 Table). In wild jujube, zinc finger proteins were the most abundant, followed by MYB, ERF, bHLH, WRKY (Table 3). In this present study, The WRKY family had the most abundant DEGs (29), followed by ERF (27), and zinc finger (21). Meanwhile, the percentage of DEGs in the WRKY family was also the highest (33.33%), followed by DREB (23.81%) and ERF (22.13%) (Table 3). MYB, bHLH, and NAC were also highly enriched in the alkaline stress treatments (Table 3).

**Table 3:** The transcriptional factors identified from the six transcriptomes of wild jujube roots after 1 h or 12 h alkaline treatment.

TF family	Total TFs	Differentially expressed TFs number			
		Total	1 h	12 h	Percentage (%)
ERF	122	27	15	20	22.13
bHLH	102	17	6	15	16.67
DREB	21	5	2	5	23.81
WRKY	87	29	15	28	33.33
MYB	151	18	4	17	11.92
NAC	75	10	3	10	13.33
Zinc finger	284	21	8	18	7.39
bZIP	58	1	1	0	1.72

#### Serine/threonine-protein kinase (STPK)

**Fig 5:**
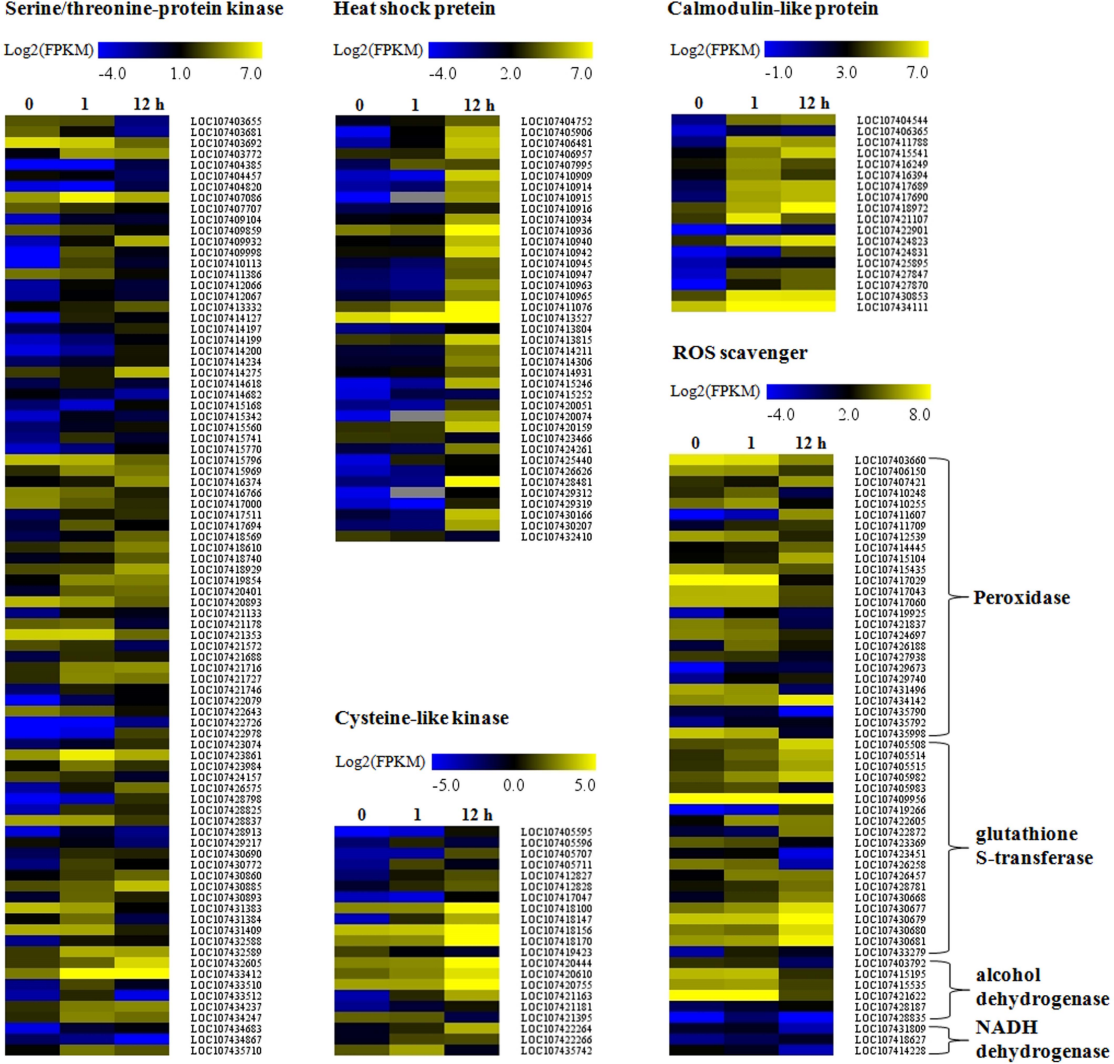
Expression profile of the different family genes in response to alkaline stress at different time points. The heat map was constructed based on log_2_ FPKM values. The color scales are shown at the top of each analysis.

We identified 39 and 59 DEGs of the STPK family after alkaline stress for 1 h and 12 h, respectively, which presented 86 DEGs among 0, 1, and 12 h (Fig 5 and S9 Table). Most DEGs (38/39) were induced by alkaline stress at 1 h (Fig 5 and S9 Table).

#### Heat shock proteins (HSP)

We identified 39 unique HSPs including HSP70s and small HSPs, which were differentially expressed under alkaline stress (Fig 5 and S9 Table). Among them, 37 genes (94.87% in DEGs) were strongly induced during alkaline stress, while the other two HSPs were inhibited at 12 h.

#### Cysteine-rich receptor-like protein kinases (CRK)

In the present study, 21 DEGs from the CRK family were identified, and most of the DEGs (18/21) were highly induced by alkaline stress (Fig 5 and S9 Table).

#### Calmodulin-like (CML) proteins

In the present study, we identified 18 CMLs that were responsive to alkaline stress, and all the 18 CMLs were activated by alkaline stress (Fig 5 and S9 Table).

#### ROS scavenger

In this study, dozens of genes appear to protect plant cells from oxidative damage by scavenging ROS, such as a batch of peroxidase genes, glutathione S-transferase genes, genes encoding alcohol dehydrogenase proteins, and NADH dehydrogenase [16–18]. For peroxidase genes, 11 of the 26 DEGs were up-regulated, and the other 15 genes were down-regulated. Besides the peroxidase gene family, a number of glutathione S-transferase genes were differentially expressed. Most of these genes (16/20) were up-regulated during alkaline stress. In addition, a few differentially expressed genes encoding alcohol dehydrogenase and NADH dehydrogenase were also identified (Fig 5 and S9 Table).

## Discussion

### Transcriptional factors (TFs) involved in alkaline stress

TFs are key regulators of gene expression, and have a variety of important functions in plant development and response to abiotic stress [19]. Therefore, identification and characterization of TFs involved in abiotic stress response is crucial to reveal the molecular mechanisms. In this study, many TFs were differentially expressed under alkaline stress (S8 Table), in that WRKY, DREB, ERF, MYB, bHLH, and NAC were highly enriched in the alkaline stress treatments (Table 3). Similarly, previous studies have shown that WRKY, ERF, EREB, MYB, bHLH, and NAC are enriched in *T*. *hispida* and *Glycine soja* roots under NaHCO_3_ stress [9,20]. However, in present study, only one member of bZIP family was differentially expressed during stress. In *T*. *hispida* roots, only two DEGs from the bZIP family were identified under NaHCO_3_ stress [9]. These results suggested that WRKY, ERF, DREB, MYB, bHLH, and NAC families were more closely related to alkaline stress in plants than bZIP. These TFs were highly enriched under alkaline stress, suggesting that they are involved in the regulation of alkaline tolerance.

### Serine/threonine-protein kinase (STPK) associated with alkaline stress

There are a series of complex regulatory mechanisms in plants that enable them to adapt to various environmental stresses. Among these mechanisms, phosphorylation by protein kinase is one of the central signaling events that occurs in response to environmental stresses [21]. Typically, eukaryotic protein kinases have been subdivided into three categories, serine/threonine protein kinase (STPK), tyrosine protein kinase (TPK), and histidine protein kinase (HPK). STPK is involved in abiotic stress, such as salt, drought, and cold, and has been reported in peanut [22], alfalfa [23], Arabidopsis [24], rice [25], and wheat [26]. Overexpression of a STPK family gene *AtSnRK2*.*8* enhances drought tolerance in Arabidopsis [24]. In rice, Overexpression of *OsSAPK4* significantly enhances the salt tolerance of transgenic plants [25]. Ectopic expression a STPK gene *TaSnRK2*.*4* of wheat resulted in longer primary roots, delayed seeding establishment, and enhanced tolerance to abiotic stresses in Arabidopsis [26]. In this study, we identified 39 and 59 DEGs of the STPK family after alkaline stress for 1 h and 12 h, respectively (Fig 5 and S9 Table). Furthermore, most DEGs (38/39) were induced by alkaline stress at 1 h (Fig 5). The fact that a large number of STPK genes were highly induced indicates its important role in signal transduction at the early stage of alkaline treatment.

### Heat shock proteins (HSPs) were highly induced

The heat shock protein (HSP) superfamily is one of the most ubiquitous and evolutionarily conserved groups across all eukaryotic species. The main function of HSPs is chaperone activity, which can stabilize chromatin structure, membranes, and unfolded proteins through the refolding of proteins under environmental stress [27, 28]. On the basis of their molecular weight, the HSP family has been classified into five groups: HSP100s, HSP90s, HSP70s, HSP60s, and small HSPs (sHSPs, 12–40 kDa) [29]. Small HSPs (sHSPs), which are especially abundant in plants, play important roles in abiotic stress tolerance and are found in great abundance and diversity [30]. Overexpression of HSPs are known to confer tolerance to thermal, oxidative, saline, osmotic, and drought stress [30–32]. For example, Overexpression a small heat shock protein, RcHSP17.8, of *Rosa chinensis* confers resistance to a variety of stresses to *Escherichia coli*, yeast and *Arabidopsis thaliana* [30]. A small heat shock protein AtHSP21 coupled with plastid metalloprotease FtsH6 regulate thermomemory in Arabidopsis [31]. Overexpression of *MuHSP70* gene from *Macrotyloma uniflorum* confers multiple abiotic stress tolerance in transgenic *Arabidopsis thaliana* [32]. Our observation that the orthologous of RcHSP17.8 and AtHSP21 in wild jujube, LOC107410945 and LOC107414931, which were both up-regulated during alkaline stress. Furthermore, we identified 39 DEGs, which belong to HSPs family (Fig 5 and S9 Table). Among them, 37 genes (94.87% in DEGs) were strongly induced during alkaline stress, while the other two HSPs were inhibited at 12 h. A batch of HSPs were induced by alkaline stress, suggesting that HSPs play significant roles in plant alkaline tolerance.

### Cysteine-rich receptor-like protein kinases (CRKs) involved in alkaline stress tolerance

When plants are exposed to abiotic stresses, external signals are likely first sensed by receptors localized in the membrane, and then downstream factors perceive the signals and activate multiple reactions to defend stress. Receptor-like kinase (RLK) may be the first sensor in this signal transduction process. RLKs play important roles in plant development and defense responses. Cysteine-rich receptor kinases (CRKs) belong to a subgroup of RLKs family which are characterized by one or more repeats of unknown function 26 (DUF26) domains consisting of a C-X8-C-X2-C motif. CRKs have been reported to positively regulate abiotic or biotic stress tolerance [33–35], and be involved in hormone perception [36]. In Arabidopsis, there are more than 40 CRKs. To date, several CRKs have been isolated and characterized. Overexpression of *AtCRK13* results in enhanced resistance to *Pseudomonas syringae* [33]. *AtCRK6* and *AtCRK7* function in protecting against apoplastic oxidative stress [34]. Overexpression of *AtCRK5* enhances abscisic acid sensitivity and confers drought tolerance in Arabidopsis [35]. In wild jujube, little is known about the function of CRKs. In the present study, 21 DEGs from the CRK family were identified, and most of the DEGs (18/21) were highly induced by alkaline stress (Fig 5 and S9 Table), suggesting that CRKs play important roles in defense against alkaline stress in the roots of wild jujube.

### Calmodulin-like (CML) proteins are highly activated

Calcium (Ca^2+^), a secondary messenger, plays an important role in plant stress responses [37], and the Ca^2+^ signals are perceived by various Ca^2+^ sensors, including calmodulins (CaM), calmodulin-like proteins (CMLs), calcium dependent protein kinases (CDPKs) and calcineurin B-like proteins (CBLs) [38,39]. In Arabidopsis, certain CMLs have been functionally characterized and it was found that some CLMs, such as CML9, CML37 were involved in abiotic stress in plant [40,41]. *AtCML9* positively regulate plant responses to abiotic stress and abscisic acid in Arabidopsis [40]. The up-regulated *CML9* (LOC107425895) in wild jujube might contribute to alkaline tolerance. AtCML37, also functions as a positive regulator in Ca^2+^ signaling during herbivory, connecting Ca^2+^ and jasmonate signaling [41]. Our observation that LOC107427847, an ortholog of AtCML37, was up-regulated by alkaline stress indicate that LOC107427847 might function in Ca^2+^ signaling during alkaline treatment. Recently, ectopic expression of *GsCML27* from *G*. *soja* enhanced plant tolerance to bicarbonate stress in *Arabidopsis* [42]. In our results, the CML27(LOC107434111) in wild jujube was also up-regulated during alkaline treatment. Furthermore, in the present study, we identified 18 CMLs that were responsive to alkaline stress, and all the 18 CMLs were activated by alkaline stress (Fig 5 and S9 Table), indicating that CMLs were closely involved in signal transduction under alkaline stress. To characterized the function of CMLs of wild jujube in alkaline stress will be carried out in next stage.

### Genes related to ROS scavenging were differentially expressed during alkaline stress

It has been reported that ROS has a dual role in plant biology as both key regulators of growth, development, stress-induced signal transduction and toxic byproducts of aerobic metabolism [43,44]. On the one hand, ROS as a signal molecule, play a key role in regulating gene expression. On the other hand, Over-accumulation of ROS cause harmful effects to plant. Previous studies have uncovered some of the key components involved in the ROS signal transduction pathway of plants, such as unidentified receptor proteins; serine/threonine-protein kinase (STPK); heat shock proteins (HSP); calmodulin-like (CML) proteins [45–48]. These molecular components play important roles in ROS sensing of plant cell. In our study, we found that a batch of STPK, HSP, CML were activated at the early stage of alkaline treatment (1 h). As a signal molecule, ROS also activated several TFs members, such as MYB, WRKY, ERF, Zinc finger families [49–52]. Consistent with previous reports, in present study, many MYB, WRKY, ERF, Zinc finger members were strong induced by alkaline stress.

Previous studies showed that abiotic stress, such as drought and salt stresses, induce ROS accumulation [43,53]. Over-accumulation of ROS usually results in oxidative stress, which cause cellular damage and death [54]. In this study, We found that alkaline stress induced H_2_O_2_ significant accumulation (Fig 1B). Accompanied by the H_2_O_2_ over-accumulation, the root system activity was decreased quickly (Fig 1C). Plant roots are the primary organ to perceive abiotic stresses such as drought, salt or alkaline. In present study, alkaline stress induced oxidative stress, which caused cell death of seedlings roots and then resulted in impairing root system activity. Therefore, a key problem that plants grown under abiotic stress face is how to weaken oxidative harmful effects. When exposed to oxidative stress, plant adopted three lines to defend against oxidative stress: (i): avoidance of ROS production; (ii): detoxification of ROS; (iii): Repair of ROS-mediated damage [16]. In this study, dozens of genes appear to protect plant cells from oxidative damage by scavenging ROS, such as a batch of peroxidase genes, glutathione S-transferase genes, genes encoding alcohol dehydrogenase proteins, and NADH dehydrogenase [16–18]. A batch of peroxidase and glutathione S-transferase genes were up-regulated during alkaline stress. In addition, several genes encoding alcohol dehydrogenase and NADH dehydrogenase were also induced (Fig 5 and S9 Table). These data suggested that there was a mechanism specifically regulating ROS homeostasis under alkaline stress.

## Materials and methods

### Plant materials, growth conditions and stress treatments

Seeds from one accession of wild jujube were germinated on blotter paper at 28°C and transferred to an artificial climate chamber (light intensity 12,000 lx, relative humidity of 65–70%, 14 h light, 10 h dark, and an average temperature of 28°C). Once seedlings had two cotyledons, they were cultured in Kimura’s culture solution B with the following composition: 0.18 mM (NH4)_2_SO_4_, 0.27 mM Mg(SO_4_)_2_, 0.091 mM KNO_3_, 0.091 mM KH_2_PO_4_, 0.046 mM K_2_SO_4_, 0.18 mM Ca(NO_3_)_2_, and 0.04 mM EDTA-Fe, and grown until the six-leaf stage.

For the alkaline treatment, seedlings of six-leaf stage were transferred to Kimura’s culture solution B, and the solution was adjusted to pH 9.5 by adding 25 mM NaHCO_3_ and 5 mM NaOH. Solutions were renewed every 3 d. Seedlings were exposed to alkaline stress of pH 9.5 for 0, 1, and 12 h, respectively. Following the treatments, roots from three seedlings of each group were harvested and pooled for RNA-seq and quantitative real-time PCR (qRT-PCR) analysis. The harvested roots were frozen in liquid nitrogen and stored at -80°C prior to RNA extraction.

### H_2_O_2_ content and root activity measurement

The level of H_2_O_2_ was assessed as described in previous study [12]. Briefly, 200 mg of fresh leaf tissue of wild jujube seedlings subjected to alkaline treatment of pH 9.5 for 0, 3, 5 days was extracted in 2.0 ml of TCA (0.1% w/v) on ice, and the homogenate was then centrifuged at 12,000 rpm for 10 min. After the addition of 0.3 mL of 10 mM sodium phosphate buffer (pH 7.5) and 0.6 mL of 1 M potassium iodide to 0.3 mL of the above supernatant, the absorbance of the samples was measured at 390 nm. H_2_O_2_ content, expressed as nmol g^-1^ fresh weight, was determined based on the standard curve generated from known concentrations of H_2_O_2_ (https://www.protocols.io/view/h2o2-content-measurement-jqhcmt6).

The activity of the root system was determined as described by Yang et al. [55]. 0.5 g of fresh root of wild jujube seedlings subjected to alkaline treatment of pH 9.5 for 0, 3, 5 days was incubated in 10 mL triphenyl tetrazolium chloride (TTC) solution (0.04% in pH 7.0 phosphate buffer) for 1 h at 37°C. After the addition of 2 mL 1M H_2_SO4, the red product in root was extracted using ethyl acetate. The absorbances were determined by spectrophotometer at 485 nm. The activity of the root system was expressed relative to the control value of 100% (https://www.protocols.io/view/root-activity-measurement-jqncmve).

### RNA extraction and purification

Total RNA was extracted using MiniBEST Universal RNA Extraction Kit (TaKaRa, Dalian, China) following the manufacturer’s instructions, and checked for an RIN number to inspect RNA integrity by an Agilent Bioanalyzer 2100 (Agilent Technologies, Santa Clara, CA, USA). Qualified total RNA was further purified by RNA Clean XP Kit (Beckman Coulter, Brea, CA, USA) and RNase-Free DNase Set (QIAGEN, GmBH, Germany).

### Illumina sequencing

The cDNA library was constructed and sequenced by Shanghai Biotechnology Corp. (Shanghai, China). Briefly, the poly(A) mRNA was enriched via magnetic oligo (dT) beads and then broken into short fragments using an RNA Fragmentation Kit (Beckman Coulter, Brea, CA, USA). These cleaved mRNA fragments were used as templates for first-strand cDNA synthesis using the random hexamer primers and Superscript II reverse transcriptase (Invitrogen, Carlsbad, CA, USA). Then, second-strand was synthesized using Invitrogen DNA polymerase 1 (Invitrogen, Carlsbad, CA, USA). After performing end repair and poly-adenylation, the mRNAs were ligated to adapters before PCR amplification. The enriched cDNA templates that were 150 nucleotides (nt) long were purified and used for further analysis. The libraries were qualified using a Qubit dsDNA HS Assay Kit (Invitrogen, Carlsbad, CA, USA) and Agilent High Sensitivity DNA Kit (Agilent Technologies). The purity and size of the libraries were checked by Qubit 2.0 Fluorometer and Agilent 2100 Bioanalyzer (Agilent Technologies). Finally, the adapter-ligated cDNA fragment libraries were run on an Illumina GAIIx analyzer to complete the cluster generation and primer hybridization. Then, the Illumina PE flow cell (v3-HS) carrying clusters were sequenced with paired-end 2×100 nt multiplex on an Illumina HiSeq 2500 platform following the manufacturer’s instruction (Illumina, San Diego, CA, USA). The transcriptome datasets were deposited at the NCBI SRA database under accession numbers SRP093586.

### Sequence assembly and annotation

After sequencing, image data were transformed into raw reads and stored with a FASTQ format. Raw reads were cleaned by removing adapter sequences, low quality at 3' end with Q < 20 (Q = -10log(error_ratio), rRNA, sequences shorter than 25 nt. The genome sequence of *Zizyphus jujuba* retrieved from the NCBI Database (http://www.ncbi.nlm.nih.gov/genome/) was used as the reference database. All clean reads were then mapped to the reference genome using TopHat2 (v. 2.0.9) [56]. The expression levels of the mapped genes were estimated from the transcriptome sequencing data based on the number of clean reads. The number of reads mapped to each gene were counted by HTSeq (v. 0.6.1) [57]. Transcript abundance was normalized by the FPKM (fragments per kilobase of exon per million fragments mapped) using Cufflinks (v. 2.1.1) [58].

### Identification of differentially expressed genes (DEGs)

To identify differentially expressed genes (DEGs), the bioconductor package edgeR (v. 3.0.8) was used to identify differential transcript accumulation between two samples[59]. The false discovery rate (FDR) was used to determine the threshold of the *P* value in multiple tests and analyses. In this study, a modified *P* value (*Q* value) ≤ 0.05 and a |log_2_(fold change)| ≥ 2 were used to recognize the significance of the gene expression difference. The heat map for DEGs were generated using Multi Experiment Viewer (MeV4.9.0) software package [60].

### Functional annotation

Functional enrichment of the DEGs was analyzed using the web-based tools in DAVID (v. 6.8) to identify enriched gene ontology (GO) terms and Kyoto Encyclopedia of Genes and Genomes (KEGG) pathways [61]. GO functional enrichment and KEGG pathway enrichment with *Q* ≤ 0.05 were taken into account as significantly enriched among the DEGs.

### Quantitative real-time PCR (qRT-PCR) analysis

Total RNA was extracted from the roots of seedlings. For qRT-PCR, the first-strand cDNA was synthesized from 1 μg total RNA according to the manufacturer’s protocol (Promega, Madison, USA). The qRT-PCR analysis was performed with the SYBR Green I Master according to the manufacturer’s protocol, and with three technical replicates for each gene. For normalization, the *ZaActin* gene (Genebank accession number, KT381859) was used as the endogenous. The relative quantification method (2^-ΔΔCT^) was used to evaluate quantitative variation between replicates examined [62]. Primers used for qRT-PCR were listed in S10 Table.

## Conclusions

In conclusion, the transcriptomes of wild jujube under alkaline stress for 0, 1, and 12 h were monitored, and 3,604 stress-responsive genes were identified. These DEGs included transcriptional factors, serine/threonine-protein kinases, heat shock proteins, cysteine-rich receptor-like protein kinases, calmodulin-like proteins, and genes related to ROS scavenging. All of these DEGs exhibited complex and diverse mechanisms under alkaline stress. Roots are the primary organ for carrying mineral nutrients and water to the rest of the plant, and also the primary site of the perception salt or alkaline stress. Therefore, this study on how the transcriptomes of roots respond to alkaline stress will enrich our knowledge of the stress tolerance of wild jujube at the molecular level and provide new insight to better understand the alkaline stress tolerance in plants.

## Supporting information

S1 Table List DEGs in the data set (0 vs. 1 h). (XLSX)

S2 Table List DEGs in the data set (0 vs. 12 h). (XLSX)

S3 Table List DEGs in the data set (1 h vs. 12 h). (XLSX)

S4 Table List DEGs in the three data sets. (XLSX)

S5 Table Gene ontology classification in the three data sets. (XLSX)

S6 Table KEGG classification in the three data sets. (XLSX)

S7 Table List of top 10 pathways of DEGs in the three data sets. (XLSX)

S8 Table List of TFs involved in alkaline stress. (XLSX)

S9 Table List of main family genes involved in alkaline stress. (XLSX)

S10 Table Primer used for quantitative real-time PCR. (XLSX)
